# Comparison of clinical characteristics between SARS-CoV-2 Omicron variant and Delta variant infections in China

**DOI:** 10.3389/fmed.2022.944909

**Published:** 2022-10-12

**Authors:** Qinggang Li, Xiaorui Liu, Lei Li, Xiaobo Hu, Guangying Cui, Ranran Sun, Donghua Zhang, Juan Li, Yonghong Li, Yong Zhang, Shen Shen, Ping He, Shasha Li, Yanmin Liu, Zujiang Yu, Zhigang Ren

**Affiliations:** ^1^Department of Infectious Diseases, The First Affiliated Hospital of Zhengzhou University, Zhengzhou, China; ^2^Precision Medicine Center, Gene Hospital of Henan Province, The First Affiliated Hospital of Zhengzhou University, Zhengzhou, China; ^3^Anyang City Fifth People’s Hospital, Anyang, China

**Keywords:** coronavirus disease 2019 (COVID-19), Omicron variant, Delta variant, severe acute respiratory syndrome coronavirus 2 (SARS-CoV-2), clinical characteristics

## Abstract

**Background:**

The continued ‘evolution’ of Severe acute respiratory syndrome coronavirus 2 (SARS-CoV-2) has led to the emergence of the Omicron variant after the Delta variant, resulting in a significant increase in the number of people with COVID-19. This increase in the number of cases continues to have a significant impact on lives. Therefore, a more detailed understanding of the clinical characteristics of Omicron infection is essential.

**Methods:**

Using medical charts, we extracted clinical information for 384 patients infected with the Omicron variant in Anyang City, Henan Province, China. Epidemiology and clinical characteristics were compared with a cohort of people infected with the Delta variant in Zhengzhou in 2021.

**Findings:**

Common initial symptoms at onset of illness were cough [240 (63%)], expectoration [112 (29%)], fever [96 (25%)], nasal congestion [96 (25%)] and myalgia or fatigue [30 (6%)]. In patients with the Omicron variant, levels of total cholesterol, low-density lipoprotein and creatinine increased in 52 (14%), 36 (9%) and 58 (15%) patients, respectively, compared with patients with the Delta variant [one (1%), one (1%) and two (2%)]. Levels of triglyceride and high-density lipoprotein also increased. In patients with the Omicron variant, the levels of specific gravity and the erythrocyte sedimentation rate were increased in 115 (30%) and 81 (21%) patients, and serum levels of complement 3 decreased in 93 (41%).

**Results:**

Compared with patients infected with Delta, no major differences in initial clinical symptoms were identified in patients infected with Omicron. However, dyslipidemia and kidney injury were much more severe in patients with the Omicron variant, and the erythrocyte sedimentation rate was increased. Due to decreased levels of complement 3, the immunity of patients with the Omicron variant was weak.

## Introduction

Coronavirus disease 2019 (COVID-19) caused by severe acute respiratory syndrome coronavirus 2 (SARS-CoV-2) was first announced as a pandemic on March 11, 2020 (1). The COVID-19 pandemic has resulted in significant morbidity and mortality in both developing and developed countries and has had a huge impact on the economies and livelihoods of global society (2–4). SARS-CoV-2 is a coronavirus with the natural capacity to undergo mutation and antigenic variation over time (5). For example, it has been reported that the increased transmissibility of the Delta variant is associated with higher viral loads, longer duration of infection, and high rates of reinfection because of its high immune escape ability (6–8). On November 26, 2021, a novel variant was reported after being named Omicron (B.1.1.529) and identified by the World Health Organization (WHO) as a variant of concern (VOC) (9). It was first identified in South Africa in November 2021, and early studies showed that infection with Omicron is associated with significantly reduced severity and mortality compared with COVID-19 caused by previous variants (10, 11). Serum from people vaccinated with two doses of mRNA or vector vaccine enabled neutralization of Omicron variant to a lesser extent than of the Delta variant (12). Overall, the Omicron variant is probably more infectious than the Delta variant (13). Compared with the original wild-type strain of SARS-CoV-2, Omicron carries up to 30 single point mutations, three deletion mutations, and one insertion mutation of the spike protein, which is the target of most therapeutic antibodies and COVID-19 vaccines (14). This high variability is reflected in diverse behaviors, with the Omicron variant showing antibody evasion, vaccine resistance, and enhanced transmission (15, 16). Furthermore, available evidence suggests that recovered patients remain susceptible to Omicron, with the occurrence of immune escape and breakthrough reinfection (17, 18). However, current studies have demonstrated that the viremic effect of Omicron is milder than previous variants or the original virus (19, 20). As of May 9, 2022, more than 515 million individuals had laboratory-confirmed SARS-CoV-2 Omicron VOC infection globally; there have also been more than 6.25 million deaths, and these numbers continue to rise rapidly (21). Therefore, greater knowledge of SARS-CoV-2 Omicron infection is urgent.

On January 8, 2022, two patients were confirmed to be infected with SARS-CoV-2 in Anyang, Henan Province, China, and a new wave of COVID-19 infections ensued. Whole-genome sequencing of the virus showed that both local cases involved infection with the Omicron variant strain BA.1 branch. After epidemiological investigation and genetic sequencing, the first outbreak in Henan was found to be homologous to that in Tianjin. To thoroughly identify potential population infections through multiple nucleic acid tests and close contact tracing and isolation, a group of patients were diagnosed with Omicron variant infection by laboratory confirmation and sent to designated hospitals for centralized treatment. Hence, these patients provide an essential opportunity to understand the clinical characteristics, laboratory findings, treatment and prognosis of the Omicron variant.

We also collected clinical statistics for patients infected with the Delta variant in October 2021. In our study, patients infected with the Delta variant in 2021 and the Omicron variant of the present outbreak were compared to explore differences in clinical characteristics to better understand the characteristics of the SARS-CoV-2 Omicron variant.

## Materials and methods

### Data sources

On January 8, 2022, the first two cases of Omicron variant infection were reported in Henan Province, China, and given the strongly contagious and insidious nature of the variant, local nucleic acid tests were promptly carried out for the entire population. The Omicron variant had already spread insidiously for at least three generations by the time the first case was reported, causing a faster and more widespread spread. Case definitions of confirmed SARS-CoV-2 infection were in accordance with the guidelines of the National Health Commission of the People’s Republic of China. A total of 384 individuals infected with the Omicron variant admitted to the Fifth People’s Hospital of Anyang from January 8 to February 13 were enrolled as the Omicron variant cohort. We collected information on patients admitted to the designated treatment hospital with laboratory-confirmed SARS-CoV-2 Omicron variant infection, including dates of illness onset, close contact with confirmed or suspected Omicron variant cases, and hospital admissions. Epidemiological data were collected through brief interviews with each patient. Clinical data for 103 patients infected with the Delta variant were collected in Zhengzhou from 30 July 2021 to 30 November 2021.

This study was approved by the Institutional Review Board of the First Affiliated Hospital of Zhengzhou University (L2021-Y429-002). The study was implemented in accordance with the Helsinki Declaration and Rules of Good Clinical Practice. Due to the urgent need to collect data on this emerging pathogen, the requirement for informed consent was waived. By extracting the medical records of patients, we used a standard case report form to gather clinical data. If the details were unclear, the working group in Zhengzhou contacted the doctors responsible for patient treatment for clarification.

### Laboratory confirmation

Cases of COVID-19 were diagnosed by detecting SARS-CoV-2 RNA in a combined nasopharyngeal and oropharyngeal swab or sputum by real-time reverse transcription–polymerase chain reaction (RT–PCR). The collection of nasopharyngeal swabs was performed by well-trained medical staff in the same hospital, and the standardized procedures were strictly followed. The samples were stored in virus medium. Viral RNA was extracted within 2 h using the Q1Aamp Viral RNA Mini Kit (Beijing Kinghawk Pharmaceutical Co., Ltd.) according to the manufacturer’s instructions. RT-PCR was performed by using the RNA Detection Kit for SARS-CoV-2 (Beijing Kinghawk Pharmaceutical Co., Ltd.) subsequently. RT-PCR was conducted with specific primers (ORF1ab-F: TGGTACTGGTCAGGCAATAAC; ORF1ab-R: TGATCTATGTGGCAACGGC; N-F: GACCCCA AAATCAGCGAAATG; N-R: CCACTGCGTTCTCCATTCTG) and Taqmen probes (ORF1ab-P: CTTTGGTGGTGCAT CGTGTTGTCT; N-P: TGCCAGTTGAATCTGAGGGTCCAC) targeting at the N, ORF1a/b genes and a positive reference gene. Reaction system and amplification conditions were performed according to the manufacturer’s specification (Beijing Kinghawk Pharmaceutical Co., Ltd.).

The detection limit of cycle threshold (Ct) was set to be 38.0 (500 copies/ml). Samples with Ct of less than 38.0 were considered positive. The cut-off Ct value of 38.0 was determined *via* the receiver operating characteristics (ROC) curve method. All tests were performed under strict biosafety conditions and the standard operating procedures.

According to sequencing viral RNA gene and comparing the results with known viral reference sequences, it was found that all patients were infected with Omicron variant. Laboratory tests, together with a whole blood count and serum biochemistry, were conducted on admission to the hospital.

### Definitions

The date of diagnosis was defined as the date of first positive nasopharyngeal swab collection. The incubation period was defined as the date from exposure to the onset of illness, which was estimated for patients who could provide the exact date of close contact with individuals with confirmed or suspected SARS-CoV-2 Omicron variant infection. Patients were discharged when their respiratory symptoms and chest CT images improved and when nasopharyngeal and oropharyngeal swab specimens collected at least 24 h apart were negative by two consecutive real-time reverse transcription-polymerase chain reaction tests.

### Statistical analysis

Categorical variables are described as percentages. For continuous variables, we calculated either means and standard deviations or medians with interquartile ranges. Means of continuous variables were compared using independent Group *t* tests when the data were normally distributed; otherwise, the Mann–Whitney test was used. Proportions for categorical variables were compared using the chi-square test; Fisher’s exact test was used when the data were limited. Differences were statistically significant when the 2-sided P was less than 0.05. SPSS software for Windows, version 26 was used for statistical analysis.

## Results

### Epidemiological characteristics

Clinical data for 384 patients in Henan Province with laboratory-confirmed SARS-CoV-2 Omicron variant infection were gathered up to February 13, 2022. The mean age among these patients was 26.4 years (SD ± 0.9 years; Table 1); it was 41.2 years (± 1.9) in patients infected with the Delta variant. Compared with patients infected with the Delta variant, the majority of patients in the current cohort were adolescents, accounting for 235 (61%) patients. Less than half of the 384 patients (162, 42%) were men. One hundred and sixty-six (46%) cases were associated with family clusters, which was notably different from the Delta variant cases [28 (27%)]. It is significant that 367 (95%) patients infected with the Omicron variant received at least two doses of a COVID-19 vaccine, compared with 101 (97%) patients with the Delta variant. Moreover, 97 (25%) patients could provide the exact date of close contact with someone who was confirmed or suspected to have a SARS-CoV-2 Omicron variant infection.

**TABLE 1:** Personal and clinical baseline characteristics of patients infected with the Omicron variant or Delta variant.

	Omicron variant (*n* = 384)	Delta variant (*n* = 103)	P
Age (Mean ± SD)	26.4 ± 0.9	41.2 ± 1.9	0
Age groups (years)			0
≤ 18	235 (61%)	11 (11%)	⋅⋅
19–40	72 (19%)	40 (39%)	⋅⋅
41–65	58 (15%)	39 (38%)	⋅⋅
≥ 66	19 (5%)	13 (13%)	⋅⋅
Male sex	162 (42%)	45 (44%)	0.784
Familial cluster	166 (46%)	28 (27%)	0.001
Number of vaccination doses			0
0	2 (1%)	40 (39%)	⋅⋅
1	11 (3%)	7 (7%)	⋅⋅
2	346 (90%)	53 (51%)	⋅⋅
3	21 (5%)	1 (1%)	⋅⋅
Coexisting conditions			
Any	40 (10%)	24 (23%)	0.001
Hypertension	23 (6%)	12 (12%)	0.048
Diabetes	10 (3%)	14 (14%)	0
Cardiovascular disease	6 (1.6%)	8 (8%)	0.003
Pregnancy	1 (0.3%)	1 (1%)	0.379
Malignancy	1 (0.3%)	0	1
Renal diseases	1 (0.3%)	1 (1%)	0.379
Liver disease	6 (1.6%)	3 (3%)	0.407
Fever	96 (25%)	36 (35%)	0.038
Highest temperature, °C			0.083
< 37.3	283 (74%)	67 (65%)	⋅⋅
37.3–38	48 (12%)	17 (17%)	⋅⋅
> 38	53 (14%)	19 (18%)	⋅⋅
Cough	240 (63%)	26 (25%)	0
Expectoration	112 (29%)	16 (16%)	0.005
Myalgia or fatigue	30 (6%)	9 (9%)	0.002
Nasal congestion	96 (25%)	7 (7%)	0
Loss of smell and taste	4 (1%)	2 (2%)	0.816
Headache	7 (2%)	3 (3%)	0.763
Diarrhea	6 (1.6%)	4 (4%)	0.279
Dyspnea	3 (0.8%)	0	1
Incubation period (days)	2 (1–2) (*n* = 97)	3 (2–7) (*n* = 81)	0
Time from illness onset to first hospital admission (days)	2 (1–3) (*n* = 362)	2.5 (0–5.0)	0.840
Systolic pressure, mm Hg	120 (110–125)	122 (113–130)	0.002
Heart rate	80 (75–82)	78.0 (75.0–81.3)	0.421
Respiratory rate	19 (18–20)	19 (18–20)	0.271
Oxygen saturation	99 (98–99)	97.0 (96.3–98.0)	0

Percentages do not total 100% owing to missing data.

Data are Mean ± SD, median (IQR), n (%), or n/N (%), where N is the total number of patients with available data.

P comparing Omicron variant and Delta variant are from χ^2^, Fisher’s exact test, or Mann–Whitney *U* test.

### Clinical features

Forty of the 384 (10%) patients had one or more coexisting medical conditions: 23 (6%) had hypertension, ten (3%) had diabetes, six (1.6%) had cardiovascular disease and six (1.6%) had liver disease; pregnancy, malignancy and renal disease occurred in one patient (0.3%) each (Table 1). In patients infected with the Delta variant, the most common coexisting conditions were diabetes [14 (14%)], hypertension [12 (12%)] and cardiovascular disease [eight (8%)]. The most common initial symptoms at onset of illness were cough [240 (63%)], expectoration [112 (29%)], fever [96 (25%)], nasal congestion [96 (25%)] and myalgia or fatigue [30 (6%)]. Less common symptoms were headache, loss of smell and taste and diarrhea. Only three (0.8%) patients developed dyspnea (Table 1). No major variations in initial clinical symptoms were identified among Omicron patients.

Among 97 patients who could provide the exact date of close contact with someone with confirmed or suspected Omicron variant infection, the median incubation period from exposure to symptoms was 2 days (IQR 1–2 days). According to our data, the incubation period in patients infected with the Omicron variant was significantly shorter than that in those infected with the Delta variant. The median time from illness onset to first hospital admission was also 2 days (1–3).

### Vital signs and laboratory parameters

**FIGURE 1:**
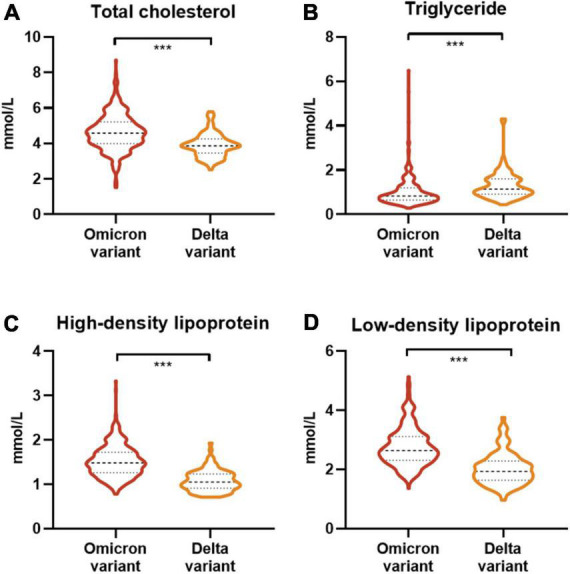
Lipid laboratory tests between patients infected with the Omicron variant or Delta variant on admission to the hospital. **(A)** Comparison of total cholesterol in patients infected with the Omicron or Delta variant. **(B)** Comparison of triglyceride in patients infected with the Omicron or Delta variant. **(C)** Comparison of high-density lipoprotein in patients infected with the Omicron or Delta variant. **(D)** Comparison of low-density lipoprotein in patients infected with the Omicron or Delta variant. The asterisk indicates a statistically significant difference compared with delta patients. *** means *P* < 0.001.

Heart rate and respiratory rate did not differ between patients infected with the Omicron variant and with the Delta variant. Compared with patients infected with Delta, systolic pressure in patients with Omicron was reduced, though oxygen saturation was increased, with both within the normal range (Table 1). These measures were recorded for all patients on the day of hospital admission. The blood counts showed leukopenia (white blood cell count less than 4 × 10^9^/L; 87 [23%] of 384 patients; Table 2). Compared with Delta-infected patients, the lymphocyte count, platelet count and hemoglobin were increased in Omicron-infected patients, and the neutrophil count was decreased. The proportion of patients with lymphopenia (lymphocyte count < 1.0 × 10^9^/L; 20 [5%] patients) was lower in the 384 patients with Omicron than in the 103 patients with Delta (15 [15%] patients). The activated partial thromboplastin time and D-dimer level were lower in Omicron cases [median activated partial thromboplastin time 30.9 s (IQR 28.7–32.9); median D-dimer level 0.04 mg/L (0.02–0.09)] than in Delta cases [33.2 s (28.9–37.3); 0.33 mg/L (0.19–0.60)]. Compared with Delta cases [12 (12%)], levels of aspartate aminotransferase were increased in 17 (4%) of the 384 Omicron cases. Moreover, some of the patients with the Omicron variant developed dyslipidemia. Levels of total cholesterol, low-density lipoprotein and creatinine in patients with the Omicron variant were increased in 52 (14%), 36 (9%) and 58 (15%) cases, respectively, and in one [1%], one [1%] and two [2%] Delta variant cases, respectively (Figures 1A,C). Compared with patients infected with Delta, levels of triglyceride and high-density lipoprotein were increased in those infected with Omicron, and lactate dehydrogenase was increased in eight (2%) (Figures 1B,C). In patients infected with the Omicron variant, levels of specific gravity and erythrocyte sedimentation rate were increased in 115 (30%) and 81 (21%) cases, respectively.

**TABLE 2:** Laboratory findings of patients infected with the Omicron variant or Delta variant on admission to the hospital.

	Omicron variant (*n* = 384)	Delta variant (*n* = 103)	P
White blood cell count, × 10^9^ per L	4.91 (4.06–6.22)	4.98 (4.00–6.21)	0.935
< 4	87 (23%)	25 (24%)	0.729
4–10	293 (76%)	76 (74%)	⋅⋅
> 10	4 (1%)	2 (2%)	⋅⋅
Neutrophil count, × 10^9^ per L	2.43 (1.82–4.33)	2.72 (2.05–3.91)	0.011
Lymphocyte count, × 10^9^ per L	1.89 (1.49–2.41)	1.54 (1.23–2.10)	0
< 1.0	20 (5%)	15 (15%)	0.001
Eosinophil count, × 10^9^ per L	0.08 (0.04–0.15)	0.08 (0.03–0.14)	0.244
Platelet count, × 10^9^ per L	205 (173–241.5)	164 (119–204)	0
> 300	18 (5%)	4 (4%)	0.935
Hemoglobin, g/L	143 (131–159)	128 (119–146)	0
Prothrombin time, s	11.7 (10.9–12.5)	11.8 (11.4–12.4)	0.071
Activated partial thromboplastin time, s	30.9 (28.7–32.9)	33.2 (28.9–37.3)	0
> 37	15 (4%)	22 (21%)	0
D-dimer, mg/L	0.04 (0.02–0.09)	0.33 (0.19–0.60)	0
≥ 1.0	2 (0.5%)	10 (10%)	0
Alanine aminotransferase, U/L	17 (12–26)	17 (12–29)	0.699
> 40	39 (10%)	26 (25%)	0.472
Aspartate aminotransferase, U/L	19 (15–24)	23 (17–30)	0
> 40	17 (4%)	12 (12%)	0.006
Albumin, g/L	43 (41.1–45.1)	42.68 (40.16–45.60)	0.203
A/G	1.6 (1.4–1.9)	1.7 (1.4–1.9)	0.186
< 1.5	123 (32%)	26 (25%)	0.184
Total Bilirubin, μmol/L	11.2 (8.48–15.03)	8.5 (6.0–12.4)	0
Total cholesterol, mmol/L	4.58 (3.99–5.21)	3.86 (3.45–4.25)	0
≥ 5.72	52 (14%)	1 (1%)	0
Triglyceride, mmol/L	0.83 (0.64–1.19)	1.14 (0.91–1.60)	0
> 2.26	11 (3%)	4 (4%)	0.833
High-density lipoprotein, mmol/L	1.48 (1.26–1.72)	1.05 (0.91–1.23)	0
< 0.91	4 (1%)	23 (22%)	0
Low-density lipoprotein, mmol/L	2.64 (2.31–3.11)	1.93 (1.64–2.29)	0
≥ 3.64	36 (9%)	1 (1%)	0.004
Creatinine, μmol/L	69 (62–78)	59.5 (49–71.5)	0
≥ 133	58 (15%)	2 (2%)	0.044
Creatinine kinase, U/L	86 (63–116)	61.5 (38.7–91.7)	0
Blood urea nitrogen, μmol/L	3.4 (3.2–4.9)	4.44 (3.82–5.21)	0.012
Lactate dehydrogenase, U/L	158 (140–180)	200 (173–243)	0
≥ 250	8 (2%)	25 (24%)	0
Specific gravity			⋅⋅
≥ 1.030	115 (30%)	NA	⋅⋅
Erythrocyte sedimentation rate, mm/h	13 (8–20)		⋅⋅
> 20	81 (21%)	NA	⋅⋅
Complement 3	0.73 (0.61–0.88) (*n* = 225)	NA	⋅⋅
< 0.7	93 (41%)	NA	⋅⋅
Complement 4	0.31 (0.25–0.44) (*n* = 225)	NA	⋅⋅
C-reactive protein, mg/L	1.57 (0.14–4.20)	3.9 (1.5–18.5)	0
> 10	28 (7%)	36 (35%)	0
Procalcitonin, ng/mL			
< 0.1	314 (82%)	95 (92%)	0.01
Pneumonia	116 (30%)	63 (61%)	0
Bilateral involvement of chest radiographs	51 (13%)	66 (50%)	0

Percentages do not total 100% owing to missing data.

Data are median (IQR), n (%), or n/N (%), where N is the total number of patients with available data.

P comparing Omicron variant and Delta variant are from χ^2^, Fisher’s exact test, or Mann–Whitney *U* test.

**FIGURE 2:**
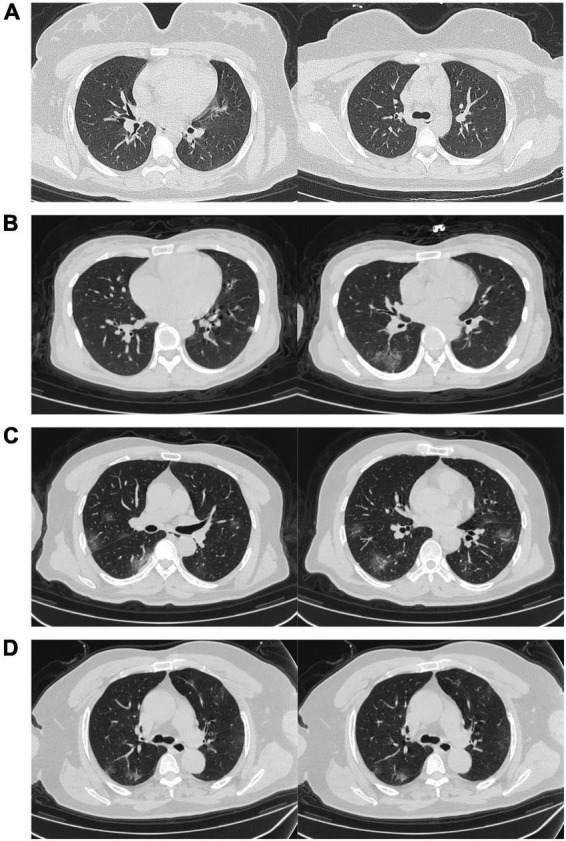
Representative chest CT images. **(A)** Transverse chest CT images for a 16-year-old female infected with Omicron only showing ground-glass opacity on day four after symptom onset. **(B)** Transverse chest CT images for a 35-year-old female infected with Omicron showing signs of bilateral pneumonia on day four after symptom onset. **(C)** Transverse chest CT images for a 51-year-old male infected with Omicron showing signs of bilateral pneumonia on day three after symptom onset. **(D)** Transverse chest CT images for a 73-year-old male infected with Omicron showing multiple bilateral pneumonia on day six after symptom onset.

On admission, serum levels of complement 3 were decreased in 93 (41%) of 225 Omicron patients. Most patients [314 (82%)] had normal serum levels of procalcitonin, and a few [28 (7%)] had abnormal levels of C-reactive protein. On admission, 116 (30%) of the 384 enrolled patients infected with Omicron developed pneumonia, and only 51 (13%) showed bilateral involvement of chest CT scans (Figure 2).

### Clinical treatment and outcomes

The complications, treatment and outcomes of the 384 patients infected with Omicron are shown in Table 3. None of these patients was transferred to an intensive care unit. Complications among the 384 patients included acute kidney injury [1 (0.3%)], anemia [1 (0.3%)] and secondary infection [1 (0.3%)]. Almost all patients [381 (99%)] received traditional Chinese medicine treatment, and 143 (37%) received immunotherapy. Only a few patients received heparin [33 (9%)], interferon inhalation [22 (6%)] or antiviral treatment [8 (2%)], with a lower proportion of use than in patients infected with the Delta variant. Furthermore, nasal cannula was required in only four (1%) patients.

**TABLE 3:** Treatments and outcomes of patients infected with the Omicron variant or Delta variant.

	Omicron variant (*n* = 384)	Delta variant (*n* = 103)	P
Admission to intensive care unit	0	10 (10%)	0
Acute kidney injury	1 (0.3%)	0	1
Anemia	1 (0.3%)	0	1
Secondary infection	1 (0.3%)	0	1
Shock	0	1 (1%)	0.211
**Treatment**			
TCM treatment	381 (99%)	93 (90%)	0
Immunotherapy	143 (37%)	35 (34%)	0.542
Antiviral treatment	8 (2%)	16 (16%)	0
Antibiotics	11 (3%)	16 (16%)	0
Interferon inhalation	22 (6%)	13 (13%)	0.016
Heparin	33 (9%)	26 (25%)	0
Chloroquine phosphate	0	3 (3%)	0.008
Glucocorticoid treatment	0	0	⋅⋅
Continuous renal-replacement therapy	0	0	⋅⋅
**Oxygen support**			
Nasal cannula	4 (1%)	17 (17%)	0
Non-invasive ventilation or high-flow nasal cannula	0	2 (2%)	0.044
Invasive mechanical ventilation	0	2 (2%)	0.044
Invasive mechanical ventilation and ECMO	0	2 (2%)	0.044
**Prognosis**			
Hospitalization	0	0	⋅⋅
Discharge	384 (100%)	103 (100%)	⋅⋅
Death	0	0	⋅⋅
Hospital stay, days	19 (15–25)	26 (18–46)	0
Disease duration, days	22 (17–28) (*n* = 362)	28 (21–47) (*n* = 101)	0

Percentages do not total 100% owing to missing data.

Data are median (IQR), n (%), or n/N (%), where N is the total number of patients with available data.

P comparing Omicron variant and Delta variant are from χ^2^, Fisher’s exact test, or Mann–Whitney *U* test.

As of April 10, 2022, all patients had been discharged from the hospital, with no deaths. In patients infected with the Omicron variant, the median time from first hospital admission to discharge was 19 days (interquartile range 15–25 days), which was shorter than that in patients with the Delta variant [26 (18–46) days]. Disease duration, the time from illness onset to discharge, was lengthened [22 (17–28) days] in patients with Omicron compared with those with Delta [28 (21–47) days].

## Discussion

As of 9 May 2022, over 220,000 laboratory-confirmed cases of infection with SARS-CoV-2 have been reported in China (22). According to a report from the WHO, the current global epidemiology of SARS-CoV-2 is characterized by continued rapid global spread of the Omicron variant. In previous studies, patients infected with the Omicron variant were less likely to be hospitalized and develop severe disease than those infected with previous variants or the original virus causing COVID-19 (23). Here, we report a cohort of 384 patients with laboratory-confirmed SARS-CoV-2 Omicron variant infection. Based on epidemiological investigation, 378 (98%) of the patients in our study had received at least two doses of COVID-19 vaccination, indicating immune escape by the Omicron variant. Common initial symptoms at onset of illness were cough, expectoration, fever, nasal congestion, myalgia and fatigue, consistent with the original wild-type strain of SARS-CoV-2 and other variant infections (19, 24). Most of the patients in Henan Province had mild symptoms, and only three patients developed dyspnea. No patients were admitted to the intensive care unit. Compared with patients infected with the Delta variant, laboratory findings showed that the patients with the Omicron variant experienced mild illness. Our results showed that most Omicron patients had levels of white blood cell counts, neutrophil counts, and lymphocyte counts in the normal range. This suggests an active interaction between immunity and the Omicron variant (19). Compared with those infected with Delta, levels of activated partial thromboplastin time were increased in patients infected with Omicron, which may be related to the increased activity of coagulation factors. There were a few patients with abnormal alanine aminotransferase, aspartate aminotransferase, lactate dehydrogenase, C-reactive protein or procalcitonin, which suggests little hepatic injury, little myocardial injury and a weak inflammatory response. Compared with patients infected with the Delta variant, the incidence of pneumonia and bilateral involvement of chest radiographs were reduced in patients infected with the Omicron variant, resulting in little pulmonary injury. Omicron infections generally cause less severe disease than infections with prior variants (25). Coexisting conditions including hypertension, diabetes and cardiovascular diseases demonstrated lower frequencies in patients with Omicron than those with Delta, which might be also associated with their younger ages. Furthermore, the significant age difference between the two cohorts may have affected the laboratory results, leading to fewer patients with mild infections than among patients infected with Delta.

Compared with those infected with Delta, levels of high-density lipoprotein were increased in patients infected with Omicron. It has been reported that low high-density lipoprotein and high triglyceride concentrations before or during hospitalization are strong predictors of COVID-19 severity (26). A higher proportion of patients with the Omicron variant had abnormally elevated total cholesterol and low-density lipoprotein than in patients with Delta. Dyslipidemia is a potential risk factor for COVID-19 (27). Studies have shown that Dyslipidaemia was associated with the severity and mortality of COVID-19 (28). A sustained inflammatory response driven by a ‘cytokine storm’, including the release of pro-inflammatory cytokines (TNF-α, IL-6, IL-8 and IL-10) and lymphopenia, is thought to be a major cause of life-threatening complications in SARS-CoV-2 patients. In the context of acute inflammation, reduced plasma levels of lecithin cholesterol acyltransferase (LCAT) may also alter HDL function and further worsen the inflammatory response (29). At the same time, LDL and its major apolipoprotein, apolipoprotein B (apoB), are oxidized (oxLDL) (30). On admission, levels of creatinine and specific gravity were increased in patients with Omicron, which indicates that Omicron variant infection might be associated with kidney injury. Similarly, a high erythrocyte sedimentation rate indicates the active stage of the disease. In addition, low serum levels of complement 3 in Omicron patients indicated reduced immunity.

Almost all patients in the two cohorts received traditional Chinese medicine treatment, but the types of drugs used varied. Because of the reduced immunity in patients with the Omicron variant, 143 (37%) received immunotherapy. In addition, the use of antiviral drugs, antibiotics and heparin was decreased in patients with the Omicron variant compared with those infected with the Delta variant. Both disease duration and hospital stay were reduced in Omicron patients. These results indicate that SARS-CoV-2 Omicron variant infection might be milder than Delta variant infection.

Given the large number of patients infected with Omicron in this study, our findings provide valuable information for understanding the clinical characteristics of Omicron variant infection. Due to the increased transmissibility and risk of reinfection of Omicron variant, it is still important to remain vigilant and protect ourselves against COVID-19.

There are several limitations in our study. First, as the patients were only from Henan Province, it is possible that more clinical features associated with Omicron variant infection were identified. Second, because of the strict control measures associated with COVID-19 in China, including epidemiological history investigation and follow-up, isolation of close contacts and early treatment of patients, patients with mostly mild disease were enrolled in our study.

## Conclusion

No major differences were identified between initial clinical symptoms in patients infected with Omicron versus those with Delta. Compared with patients infected with the Delta variant, dyslipidemia and kidney injury were much more severe in patients infected with the Omicron variant, and the erythrocyte sedimentation rate was increased. Due to decreased levels of complement 3, the immunity of patients with the Omicron variant was reduced.

## Data availability statement

The datasets presented in this article are not readily available because COVID-19 patient information is private. Requests to access the datasets should be directed to ZR, fccrenzg@zzu.edu.cn.

## Author contributions

ZY and ZR designed the study. ZY, ZR, QL, XL, XH, GC, RS, DZ, JL, YHL, YZ, SS, PH, SL, and YML treated the patients and collected the clinical data. ZR and LL analyzed the clinical data. LL and ZR wrote the manuscript. All authors reviewed and approved the manuscript.

## Abbreviations

COVID-19: coronavirus disease 2019
SARS-CoV-2: severe acute respiratory syndrome coronavirus 2
HDL: high-density lipoprotein
LDL: low-density lipoprotein
OxLDL: oxidized LDL.
